# Developing COVID-19 vaccine recommendations during the pandemic: The experience of Serbia's Expert Committee on Immunization

**DOI:** 10.3389/fpubh.2022.1056670

**Published:** 2022-11-17

**Authors:** Ljiljana Markovic-Denic, Dusan Popadic, Tanja Jovanovic, Branka Bonaci-Nikolic, Janko Samardzic, Vesna Tomic Spiric, Miljan Rancic, Siddhartha Sankar Datta, Liudmila Mosina, Jasna Jancic, Goran Vukomanovic, Verica Jovanovic, Vladislav Vukomanovic, Darko Antic, Marko Veljkovic, Vladan Saponjic, Lisa Jacques-Carroll

**Affiliations:** ^1^Faculty of Medicine, University of Belgrade, Belgrade, Serbia; ^2^Clinic of Allergy and Immunology, Clinical Center of Serbia, Belgrade, Serbia; ^3^Institute of Pharmacology, Clinical Pharmacology, and Toxicology, Belgrade, Serbia; ^4^World Health Organization Country Office Serbia, Belgrade, Serbia; ^5^World Health Organization Regional Office for Europe, Copenhagen, Denmark; ^6^Clinic for Neurology and Psychiatry for Children and Youth, Belgrade, Serbia; ^7^University Children's Hospital, Belgrade, Serbia; ^8^Institute of Public Health of Serbia, Belgrade, Serbia; ^9^Mother and Child Health Institute of Serbia, Belgrade, Serbia

**Keywords:** National Immunization Technical Advisory Group (NITAG), COVID-19 pandemic, COVID-19 vaccination, Serbia, evidence-based recommendations, immunization

## Abstract

A National Immunization Technical Advisory Group (NITAG) is a multi-disciplinary body of national experts that provide evidence-based recommendations to policy-makers to assist them in making informed immunization policy and programme decisions. During the COVID-19 pandemic, NITAGs faced many challenges in making evidence-based recommendations for COVID-19 vaccines due to the rapidly evolving situation with new vaccine products available in a short time period and limited data on vaccine effectiveness. The authors reviewed the process used by Serbia's NITAG, which is called the Serbian Expert Committee on Immunization, to develop COVID-19 vaccine recommendations during the pandemic. The article examines the challenges and successes faced by the committee. Serbia's expert committee used the best available evidence to develop over forty recommendations on all aspects of COVID-19 vaccination. These expert committee recommendations facilitated the early procurement and successful roll-out of COVID-19 vaccines, guidance for vaccination of individuals at the highest risk, and high COVID-19 vaccination coverage in the country. The availability of five COVID-19 vaccines in Serbia was an advantage for the successful roll-out but posed challenges for the expert committee. Serbia's expert committee plans to use the experience and best practices developed during the pandemic to improve and expand its work moving forward.

## Introduction

COVID-19 is a serious respiratory disease caused by SARS-CoV-2, the coronavirus that emerged in December 2019 in Wuhan, China, which eventually spread worldwide causing alarming numbers of infections, hospitalizations and deaths and overwhelming healthcare systems (1, 2). The first COVID-19 cases in Europe were reported in France on 24 January 2020. One month later, clusters of cases were detected in northern Italy (3). COVID-19 was first detected in the Republic of Serbia on 6 March 2020, shortly after the first cases in neighboring countries (4). Non-pharmaceutical interventions, especially contact tracing, were implemented at the beginning of the COVID-19 pandemic in Serbia. In Serbia, there is a long tradition of infectious disease surveillance and outbreak control performed by public health institutes (PHIs). There are 25 district PHIs and the National PHI with qualified specialists in epidemiology and other public health specialists with extensive experience in outbreak investigation and control and field epidemiology. On 15 March 2020, martial law was declared at the national level and strict measures were introduced such as restrictions on movement and gatherings, border closures, contact tracing, isolation of cases, home quarantine for close contacts, and daycare, school, and university closures. All citizens aged 65 years and older were recommended to stay indoors. A series of surveillance measures were implemented, including rapid detection of suspected cases, testing and actively identifying contacts, and cluster investigations. Martial law ended in Serbia on 6 May 2020. Since then, non-pharmaceutical measures have been continually adjusted to the prevailing epidemiological situation in the country. As of 8 July 2022, Serbia had 2,038,946 confirmed cases and 16,146 confirmed deaths linked to COVID-19 (5).

During mid-2020, in anticipation that COVID-19 vaccines under development would be available by late 2020, the Serbian Ministry of Health (MoH) recognized the essential role of their National Immunization Technical Advisory Groups (NITAG) in the development of evidence-informed national COVID-19 vaccination recommendations to introduce COVID-19 vaccines.

## Background on Serbia's Expert Committee on Immunization

### Establishment of the committee

Serbia's NITAG is called the Serbian Expert Committee on Immunization and was established by the Ministry of Health (MoH) in September 2018 (6). The role of the committee was enhanced in November 2020 in preparation for the introduction of COVID-19 vaccines.

### Committee membership and composition

The expert committee's terms of reference do not restrict the number of core members and there are currently 11 members, plus one chair. Multidisciplinary members are nominated to serve on the committee by the MoH. Before the COVID pandemic, the committee had seven members and in November 2020, new members with expertise in immunology and pharmacology were added to the committee to assist with the development of COVID-19 vaccine recommendations. Members do not have term limits and do not receive payments or per diem for their work.

Serbia's expert committee does not include ex-officio members, but the Medicines and Medical Devices Agency of Serbia (ALIMS) provides information to the committee as needed. Liaison members from medical societies representing pediatrics, gynecology, and obstetrics have been invited to committee meetings to provide opinions of their organizations during discussions about COVID-19 vaccine recommendations. World Health Organization (WHO) country office staff attend committee meetings as liaison members. The Serbian National Institute of Public Health serves as the Secretariat for the committee. Neither the public nor vaccine industry representatives are invited to attend committee meetings.

### Functioning of the committee

Serbia's MoH has developed terms of reference for the expert committee, including (1) monitoring and identifying key information on the effects, applicability, and potential barriers to the implementation of immunization programs; (2) establishing recommendations grounded on evidence-based medicine to ensure transparency and trust in the decision-making process for immunization by using reliable sources and types of information; (3) determining proposals for education on vaccination in order to increase awareness on the importance and need for vaccination; (4) defining recommendations for the promotion of disease prevention and counseling on the process of eradication of infectious diseases in order to achieve and maintain high levels of immunization coverage; (5) and defining recommendations for the adoption of public health measures related to immunization.

The expert committee normally meets quarterly, but met on an *ad hoc* basis much beyond the regular meeting frequencies during the COVID-19 pandemic. The Secretariat organizes the committee meetings, develops agendas, provides data and information, drafts meeting minutes, and finalizes the meeting report detailing expert committee recommendations. Committee members are responsible for conducting literature reviews. Agendas and information are distributed to members before committee meetings. At least half of all core members must be present for decision-making, and decisions are made by consensus.

## COVID-19 vaccine recommendations

### Developing COVID-19 vaccine recommendations

Serbia's expert committee developed over 40 recommendations on COVID-19 vaccination and all were accepted and implemented by the MoH, except the recommendation for mandatory COVID-19 vaccination for employees in health, social care and public sectors which was not accepted. Table 1 details key COVID-19 vaccination recommendations prepared by Serbia's expert committee, including recommendations on the initial priority groups, vaccination of pregnant and breastfeeding women, vaccination of children and booster doses. Both the MoH and the expert committee faced challenges using the routine approach to develop recommendations for COVID-19 vaccination due to a lack of timely evidence on vaccine product characteristics, including data on safety and efficacy, and insufficient data on disease epidemiology in the country. They also had to develop ways to cope with interruptions in routine communication methods such as restrictions on in-person meetings.

**Table 1:** Key COVID-19 vaccine recommendations developed by the Serbian Expert Committee on Immunization.

**Date**	**Recommendation**	**Main evidence considered**	**Accepted by MoH**
27 November 2020	COVID-19 vaccination of priority groups using three implementation phases	1. WHO SAGE roadmap for prioritizing the use of COVID-19 vaccines (October 2020 & November 2020) (7) 2. ECDC. Key aspects regarding the introduction and prioritization of COVID-19 vaccination in the EU/EEA and the UK (October 2020) (8) 3. JCVI. Priority groups for coronavirus (COVID-19) vaccination: advice from the JCVI (September 25, 2020) (9) 4. ACIP. Advisory Committee on Immunization Practices' Interim Recommendation for Allocating Initial Supplies of COVID-19 Vaccine—United States, 2020 (December 2020) (10)	Yes
11 January 2021	Vaccination of persons who had COVID-19: 1 month after clinical recovery for symptomatic cases and 1 month after a PCR-positive test for asymptomatic cases	1. WHO SAGE values framework for the allocation and prioritization of COVID-19 vaccination (September 2020) (11)	Yes
19 February 2021	Breastfeeding is not a contraindication for COVID-19 vaccination	1. WHO. Interim recommendations for use of the Pfizer-BioNTech COVID-19 vaccine, BNT 162b2, under Emergency use listing (January 2021) (12)	Yes
24 March 2021	COVID-19 vaccination of persons with allergic diseases, hypersensitivity reactions, and primary and secondary immunodeficiencies	1. Allergy: EAACI statement on the diagnosis, management and prevention of severe allergic reactions to COVID-19 vaccines (January 2021) (13) 2. J Allergy Clin Immunol: mRNA vaccines to prevent COVID-19 disease and reporting allergic reactions: current evidence and approach (April 2021) (14) 3. Allergol Select: Practical recommendations for the allergological risk assessment of the COVID-19 vaccination—a harmonized statement of allergy centers in Germany (January 2021) 4. Serbian Association of Allergology and Clinical Immunology: Recommendations for SARS-CoV-2 vaccination in patients with allergic diseases and hypersensitivity reactions 5. CDC. Interim Clinical Considerations for Use of mRNA COVID-19 Vaccines Authorized in the United States: people who are moderately or severely immunocompromised (March 2021) (15) 6. ESMO statements on vaccination against COVID-19 in people with cancer (December 2020)	Yes
26 April 2021	COVID-19 vaccination of pregnant women after the first trimester	1. Human Reprod Open. Joint IFFS/ESHRE statement on COVID-19 vaccination for pregnant women and those considering pregnancy (April 2021) (16) 2. New Engl J Med. Preliminary findings of mRNA COVID-19 vaccine safety in pregnant persons (April 2021) 3. Australian Government. COVID-19 vaccination decision guide for women who are pregnant, breastfeeding or planning pregnancy (March 2021) 4. Republic Expert Commission for Gynecology and Obstetrics of Serbia	Yes
	COVID-19 vaccination of persons 16–17 years of age (Pfizer-BioNTech vaccine)	1. ECDC. Interim public heath consideration for COVID-19 vaccination of adolescent in the EU/EEA (June 2021) (17)	Yes
		2. Pediatric Association of Serbia 3. Republic Experts Commission for Pediatrics	
13 May 2021	Persons who received AstraZeneca COVID-19 vaccine should receive an additional dose	WHO SAGE. Interim recommendations for the use of the ChAdOx1-S (recombinant) vaccine against COVID-19 (AstraZeneca COVID-19 vaccine AZD1222Vanzevria ^TN^, SII COVISHIELD ^TM^) (February 2021, April 2021) (18)	Yes
17 June 2021	COVID-19 vaccination of children 12–15 years of age (Pfizer-BioNTech vaccine)	1. WHO. Interim recommendations for use of the Pfizer-BioNTech COVID-19 vaccine, BNT 162b2, under Emergency use listing (January 2021, update June 2021) (12) 2. ECDC. Interim public heath consideration for COVID-19 vaccination of adolescent in the EU/EEA (June 2021) (17) 3. Pediatric Association of Serbia 4. Republic Experts Commission for Pediatrics	Yes
21 July 2021	COVID-19 booster dose at least 6 months after the second dose. For the booster, an mRNA-based vaccine is recommended, but the choice of the vaccine based on a different technology is permitted	1. BMJ. Booster vaccine to be rolled out in autumn as UK secures 60 m more Pfizer doses (April 2021) 2. JCVI interim advice: potential COVID- booster vaccine program (June 2021) (19) 3. Lancet. Tolerability and immunogenicity after a late second dose or a third dose of ChAdOx1 nCoV-19 (AZD1222; Preprint) (June 2021) (20)	Yes
4 October 2021	Additional dose of COVID-19 vaccine for persons with primary and secondary immunodeficiencies (Pfizer-BioNTech vaccine)	1. N Engl J Med. Three Doses of an mRNA COVID-19 Vaccine in Solid-Organ Transplant Recipients (June 2021) 2. ECDC. Interim public health considerations for the provision of additional COVID-19 vaccine doses (September 2021) (21) 3. WHO. Interim recommendations for use of the Pfizer-BioN COVID-19 vaccine, BNT 162b2, under Emergency use listing- (June 2021) (12)	Yes
	COVID-19 and seasonal flu vaccines can be given at the same time	1. WHO. Coadministration of seasonal inactivated influenza and COVID-19 vaccines (October 2021) (22) 2. MMWR: Prevention and control of seasonal influenza with vaccines: recommendation of the Advisory Committee on Immunization Practices, United States, 2021–2022 influenza season (August 2021)	Yes
	Heterologous COVID-19 vaccination may be considered for persons who had an allergic reaction after the first dose and <6 months have passed	1. EMA and ECDC recommendations on heterologous vaccination courses against COVID-19: “mix-and-match” approach can be used for both initial courses and boosters (December 2021) (23)	Yes
20 October 2021	Interval for COVID-19 vaccine booster dose reduced to 5 months for mRNA/viral vector vaccines and 4 months for inactivated vaccines	1. N Engl J Med: Protection of BNT162b2 vaccine booster against COVID-19 in Israel (October 2021) (24) 2. Cell Discovery: Robust induction of B and T cell responses by a third dose of inactivated SARS-CoV-2 vaccine (preprint September 2021)	Yes
	Mandatory COVID-19 vaccination for employees in health, social care, and public sectors	1. BMJ: COVID-19. Government considers mandatory vaccination for healthcare staff in England (September 2021) 2. BMJ: COVID-19: France and Greece make vaccination mandatory for healthcare workers (July 2021) (25)	No
	In hemodialysis cases with a lack of a humoral immune response after a booster dose, two doses of an mRNA vaccine should be administered 21 days apart, at least 6 weeks after the booster dose	1. EBioMedicine: Cellular and humoral immunogenicity of a SARS-CoV-2 mRNA vaccine in patients on haemodialysis (August 2021) (26)	Yes
2 December 2021	COVID-19 vaccine booster dose for children <18 years of age with primary and secondary immunodeficiencies (Pfizer-BioNTech vaccine)	1. SAGE. Highlights from the Meeting of the Strategic Advisory Group of Experts on Immunization (October 2021) (27) 2. WHO. Interim recommendations for use of the Pfizer–BioNTech COVID-19 vaccine, BNT162b2, under Emergency Use (November 2021) (12)	Yes
14 January 2022	Interval for COVID-19 booster dose reduced to 3 months, regardless of vaccine type	1. JCVI statement on the adult COVID-19 booster vaccination programme and the Omicron variant (January 2022) (28) 2. STIKO. German committee recommends booster after three months as Omicron spreads (December 2021) 3. WHO SAGE roadmap for prioritizing use of COVID-19 vaccines (January 2022) (7)	Yes
	COVID-19 vaccination of pregnant women during any trimester (Pfizer-BioNTech) COVID-19 vaccination of women prior to undergoing *in-vitro* fertilization	1. ESHRE COVID-19 Working Group. Statement on COVID-19 vaccination and assisted reproduction (January 2021) (29) 2. SOGC. Statement on COVID-19 Vaccination in Pregnancy (November 2021) (30) 3. Republic Expert Commission for Gynecology and Obstetrics of Serbia. Conclusions on vaccination against COVID-19 in pregnancy planning, pregnancy, and lactation 4. Serbian Medical Association's Gynecological and Obstetric section. Recommendations of the Working Group on vaccination against COVID-19 in planning pregnancy, pregnancy, and breastfeeding	Yes Yes
18 February 2022	Second booster dose of COVID-19 vaccine for persons 18 years and older, primarily for persons with primary and secondary immunodeficiencies, and persons over 60, at least 5 months after the first booster dose using the vaccine of their choice	1. Health Ministry, Israel: 4th dose triples protection from serious illness for over-60 (January 2022) (31) 2. Reuters. Chile, a vaccine front-runner, launches fourth COVID dose (January 2022) 3. Forbes. Denmark first in Europe to offer 4th COVID vaccine dose (January 2022) (32)	Yes
	COVID-19 vaccine booster dose for children 12–18 years of age at least 5 months after the second dose (Pfizer-BioNTech)	1. ECDC. COVID-19 vaccine effectiveness in adolescents aged 12–17 years and interim public health considerations for administration of a booster dose (February 2022) (33) 2. EMA recommends authorization of booster doses of Comirnaty from 12 years of age (February 2022)	Yes

Serbia's expert committee overcame these challenges by collecting all available evidence, primarily recommendations from leading organizations such as the World Health Organization's (WHO) SAGE (Strategic Advisory Group of Experts on Immunization) and ETAGE (European Technical Advisory Group of Experts on Immunizations), as well as ECDC (European Center for Disease Prevention and Control), United States' ACIP (Advisory Committee on Immunization Practices), United Kingdom's JCVI (Joint Committee on Vaccination and Immunization) and articles published in peer-reviewed journals and on MoH websites. WHO Regional Office for Europe provided support to Member States to develop COVID-19 vaccine recommendations through a series of webinars providing information and guidance on COVID-19 vaccines and vaccination and sharing other Member States' experiences in developing recommendations (34). In addition to continuously interpreting WHO's official positions, policies, and recommendations regarding COVID-19 vaccination, and quickly providing information on relevant policies and practices in other countries, WHO Country Office Serbia provided a connection to the WHO regional office to obtain timely advice on developing the most challenging recommendations on COVID-19 vaccination, which contributed to the successful work of Serbia's expert committee.

Each expert committee meeting was preceded by a review of all available literature by members. Joint meetings with relevant medical associations and official commissions of the MoH were organized to develop recommendations for specific groups such as children and pregnant women. External experts were involved in discussions and presented position papers and their opinions. All recommendations were evidence-based and considered local epidemiological circumstances. In making decisions, only core committee members have the right to vote but there is no formal over-voting in the case of dissenting opinions and decisions are made by consensus. At times, several meetings took place to discuss a topic until the best possible evidence was found and a consensus was reached. Once a decision was made, the committee submitted the recommendation to the Government of Serbia and the MoH, who decided whether or not to accept the recommendation.

### Evidence used to develop initial recommendations on priority groups

When developing initial recommendations on priority groups for COVID-19 vaccination, Serbia's expert committee reviewed recommendations from WHO SAGE (7, 11) and ETAGE (35), ECDC (8), JCVI (9), Israel's Advisory Committee on Infectious Diseases and Immunizations (36), ACIP (10), EMA (European Medicines Agency) (37), and considered local epidemiological data on COVID-19.

### Successes in developing COVID-19 vaccine recommendations

Serbia's expert committee met 18 times from November 2020 through February 2022 to develop recommendations on COVID-19 vaccination. Expert committee recommendations helped the MoH make informed decisions to develop national immunization policies (7). Serbia was one of the first countries in the WHO European Region to provide recommendations on the prioritization of population groups to receive COVID-19 vaccines. This facilitated timely procurement of COVID-19 vaccines and early COVID-19 vaccination in Serbia with the first vaccines administered on 24 December 2020, making Serbia the second country in the WHO European region to begin vaccinating against COVID-19, slightly later than the UK.

Some medical doctors and patients had concerns about the safety of COVID-19 vaccination for people with a history of allergic reactions (13, 14). To ensure high vaccine coverage in this group and increase confidence in vaccines among medical workers, the committee developed recommendations on how to manage patients with a history of allergic or hypersensitivity reactions including details on how to stratify the risk for allergic reactions both before the first dose of COVID-19 vaccine and for individuals who experienced reactions after COVID-19 vaccination. These recommendations were used to create algorithms for vaccinators on how to handle patients with a history of allergic reactions of different etiology as well as those who had reacted after a dose. If necessary, vaccination was preceded by consultation with an internist allergist/immunologist, and some people with allergies were vaccinated in hospitals under physician supervision. These protocols allowed individuals with a history of allergic or hypersensitivity reactions to receive COVID-19 vaccines and there were no confirmed cases of anaphylaxis in Serbia after COVID-19 vaccination. Minimizing serious adverse events following immunization (AEFIs) likely increased public confidence in COVID-19 vaccines and may have contributed to higher vaccination rates. Particular attention has been given to vaccinating people with primary and secondary immunodeficiencies (15, 26), including the administration of an additional dose (12, 21).

Serbia's expert committee provided a timely recommendation for a booster dose of COVID-19 vaccine for the most vulnerable populations based on the review of a limited number of peer-reviewed articles (19, 20), and local epidemiological data on SARS-CoV-2 transmission intensity in the country. The approach taken by the expert committee anticipated the recommendation given by ETAGE in November 2021 (38). ETAGE recommended countries make efforts to increase coverage with a primary COVID-19 vaccination series and when offering a booster dose, focus first on high-risk population groups and health care workers, which matches the recommendation made by Serbia's expert committee. The interval between completion of the primary series and the booster dose administration has been shortened several times based on new evidence and recommendations (24, 27, 28) and local data on the number of infected people.

In case of adverse events after vaccine administration, a heterologous vaccination course was accepted for both initial courses and booster doses (23). The recommendation for a second booster dose was based on evidence from several countries (31, 32) and co-administration of the inactivated influenza vaccine and COVID-19 vaccine was consistent with recommendations from WHO and others (22). Despite the committee's recommendation for mandatory COVID-19 vaccination of health care and social care workers (25), this remains voluntary Serbia.

### Collaborative mechanisms with professional associations

The expert committee collaborated with medical associations and commissions to develop recommendations for specific populations. After COVID-19 vaccines were licensed for use in children, the expert committee cooperated with the Pediatric Association of Serbia and the Republic Expert Commission for Pediatrics and initially recommended COVID-19 vaccination for adolescents aged 16–17 and later for children 12–15 years (7, 17) including a booster dose for children older than 12 years (12, 27, 33). The committee also collaborated with gynecological and obstetric commissions to develop recommendations for vaccination of pregnant women, women before undergoing *in-vitro* fertilization (16, 29, 30), and breastfeeding women (12).

### Progress in implementing COVID-19 vaccine recommendations

There was high uptake of COVID-19 vaccine by priority group 1 (residents and workers in long-term care facilities, adults 75 years and over, healthcare workers, and people with immunodeficiencies and underlying health conditions) in late December 2020. Consequently, the immunization program was able to begin vaccinating priority group 2 (individuals under 65 years of age with co-morbidities and workers in essential sectors) by January 2021.

At the beginning of the vaccine roll-out, four different COVID-19 vaccines of adequate quantity were made available in the country (Pfizer/BioNTech, Sinopharm, Sputnik V, and AstraZeneca) (12, 18, 39, 40), providing a choice of vaccine by individuals which also facilitated high vaccine uptake. In November 2021, Moderna vaccine was also introduced. Availability of various vaccine types enabled heterologous vaccination which was later found to be very efficacious in producing a robust immune response and recommended by EMA and ECDC (23).

**Figure 1:**
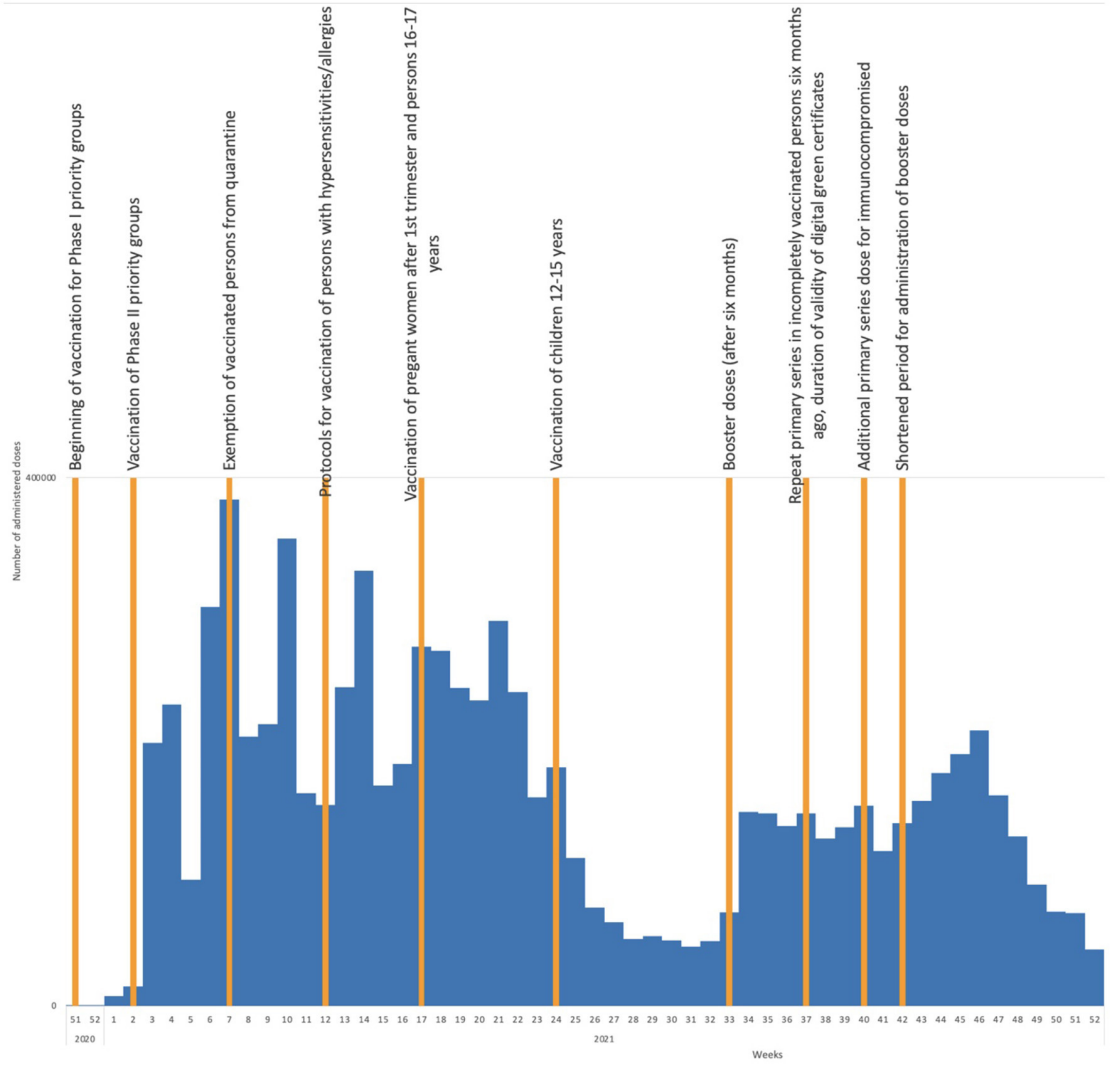
COVID-19 vaccine uptake by week in 2020 and 2021 and timing of adoption of important expert committee recommendations.

Country coverage estimates show higher COVID-19 vaccine coverage has been achieved in older age groups compared with younger age groups. As of mid-May 2022, the highest coverage with both a primary series and a booster dose was achieved in the age group of 70–79 year-olds (79.6 and 61.7%, respectively), and 60–69 year-olds (71.3 and 51.6%, respectively). The lowest coverage rates were in the age groups of 18–24 and 25–49 year-olds (27.9 and 43.5% for the primary series, and 8.7 and 19.9% for a booster dose, respectively). Coverage in adults was 54.1% for a primary series (58.0% received Sinopharm, 28.4% Pfizer/BioNTech, 9.5% Sputnik V, 4.1%, AstraZeneca, and 0.01% Moderna) and 32.7% for a booster dose (51.2% received Sinopharm, 39.6% Pfizer/BioNTech, 8.2% Sputnik V, 0.8% AstraZeneca, and 0.2% Moderna). Figure 1 shows the impact of recommendations of the expert committee on COVID-19 vaccine uptake in Serbia.

## Discussion

Serbia's expert committee provided timely recommendations on all aspects of COVID-19 vaccination which facilitated early procurement of vaccines, the successful roll-out of vaccinations, guidance for vaccination of individuals at the highest risk, and high COVID-19 vaccination coverage in the country. The MoH accepted and implemented all but one of the committee's recommendations and the committee's transparent and evidence-based process in developing recommendations improved public trust in COVID-19 vaccines. As of 18 October 2022, the WHO Regional Office for Europe COVID-19 vaccine monitor estimated uptake for a complete COVID-19 vaccine series to be 47.9% in Serbia which is quite good compared to coverage rates in the surrounding countries of Albania (44.5%), Bosnia and Herzegovina (25.8%), Bulgaria (29.8%), Croatia (55.3%), Hungary (62.9%), Montenegro (40.9%), North Macedonia (40.4%), and Romania (42.1%) (41). WHO coverage estimates are lower than Serbian estimates which is likely due to different denominators being used to measure COVID-19 vaccine coverage.

During the COVID-19 pandemic, Serbia's expert committee faced many challenges. The biggest challenge was defining vaccination priorities in a rapidly evolving situation with new vaccine products available for use within a short period of time and limited data on the effectiveness of vaccines for vulnerable population groups and others. The expert committee based its recommendations on the best available evidence from trusted sources. The regional WHO webinars were vital to learn about SAGE, ETAGE, and ECDC recommendations and to hear about other NITAG recommendations. Another significant challenge was conducting literature reviews since the Secretariat does not have staff available for this role and committee members conducted the reviews. Limited access to technical resources and scientific evidence can make it difficult to react promptly in the decision-making process (42). Open access to peer-reviewed publications through the Consortium of Serbian Libraries for Coordinated Purchase (KoBSON) and web platforms facilitated the committee's access to publications.

Serbia's expert committee benefitted from dedicated members who devoted many hours of their time reviewing and discussing evidence on SARS-CoV-2 and COVID-19 vaccines to rapidly develop evidence-based recommendations. All expert committee members were engaged in other full-time positions, including some in COVID-19 hospitals. Committee meetings were mainly held after working hours and due to the ban on group gatherings during certain stages of the pandemic, most meetings were virtual. While this was initially challenging, capacity was strengthened and the committee's functioning was successful. Despite many limitations and challenges, Serbia's expert committee used the best available evidence to develop over 40 recommendations on COVID-19 vaccination and the country achieved solid vaccination coverage in adults for a primary series and high vaccination coverage with both a primary series and a booster dose in individuals older than 60 years. Minimal AEFIs and no confirmed cases of anaphylaxis in Serbia significantly increased public confidence in COVID-19 vaccines.

There were some limitations to this study; authors could have compared the COVID-19 vaccine recommendations made by Serbia's expert committee to the recommendations made by NITAGs in other Balkan countries. In addition, if Serbian COVID-19 vaccination coverage rates were available for specific target populations (e.g., pregnant women), the authors could have measured the vaccination uptake in those groups after the expert committee issued a vaccine recommendation specific to that group to measure the impact of the recommendation.

The committee plans to use the experience and best practices developed during the pandemic to improve its work moving forward and to maintain its close working relationship with the MoH and medical commissions and societies. In the future, the committee plans to consider recommendations for routine immunizations and proposals for educational opportunities for medical staff. The committee will develop standard operating procedures for the process of evaluating evidence and developing recommendations and begin collecting declarations of interest from core members, in line with WHO recommendations (43). It will be important to expand the visibility of the expert committee by educating the public and the medical community on the process that the expert committee uses to make evidence-based immunization recommendations to increase trust in vaccines.

## Data availability statement

The original contributions presented in the study are included in the article/Supplementary material, further inquiries can be directed to the corresponding author.

## Author contributions

LM-D designed the overall concept for the article. LM-D, LJ-C, and BB-N developed the first draft of the article. MV and VS provided the data and graphics. SS and LM provided policy guidance. All authors contributed to the concept for the article, provided input and edits, and approved the final version of the manuscript for submission.

## Funding

Funding for data analysis was provided by the Serbian Ministry of Health and the National Institute of Public Health of Serbia.

## Conflict of interest

Author MR was employed by WHO Country Office Serbia. Authors SS, LM, and LJ-C were employed by WHO Regional Office for Europe. The remaining authors declare that the research was conducted in the absence of any commercial or financial relationships that could be construed as a potential conflict of interest.

## Publisher's note

All claims expressed in this article are solely those of the authors and do not necessarily represent those of their affiliated organizations, or those of the publisher, the editors and the reviewers. Any product that may be evaluated in this article, or claim that may be made by its manufacturer, is not guaranteed or endorsed by the publisher.
