# Supplementary figures and images for: Developing COVID-19 vaccine recommendations during the pandemic: The experience of Serbia's Expert Committee on Immunization
Source: Front Public Health. 2022 Nov 17;10:1056670. doi: 10.3389/fpubh.2022.1056670 (PMC9713902; doi:10.3389/fpubh.2022.1056670)

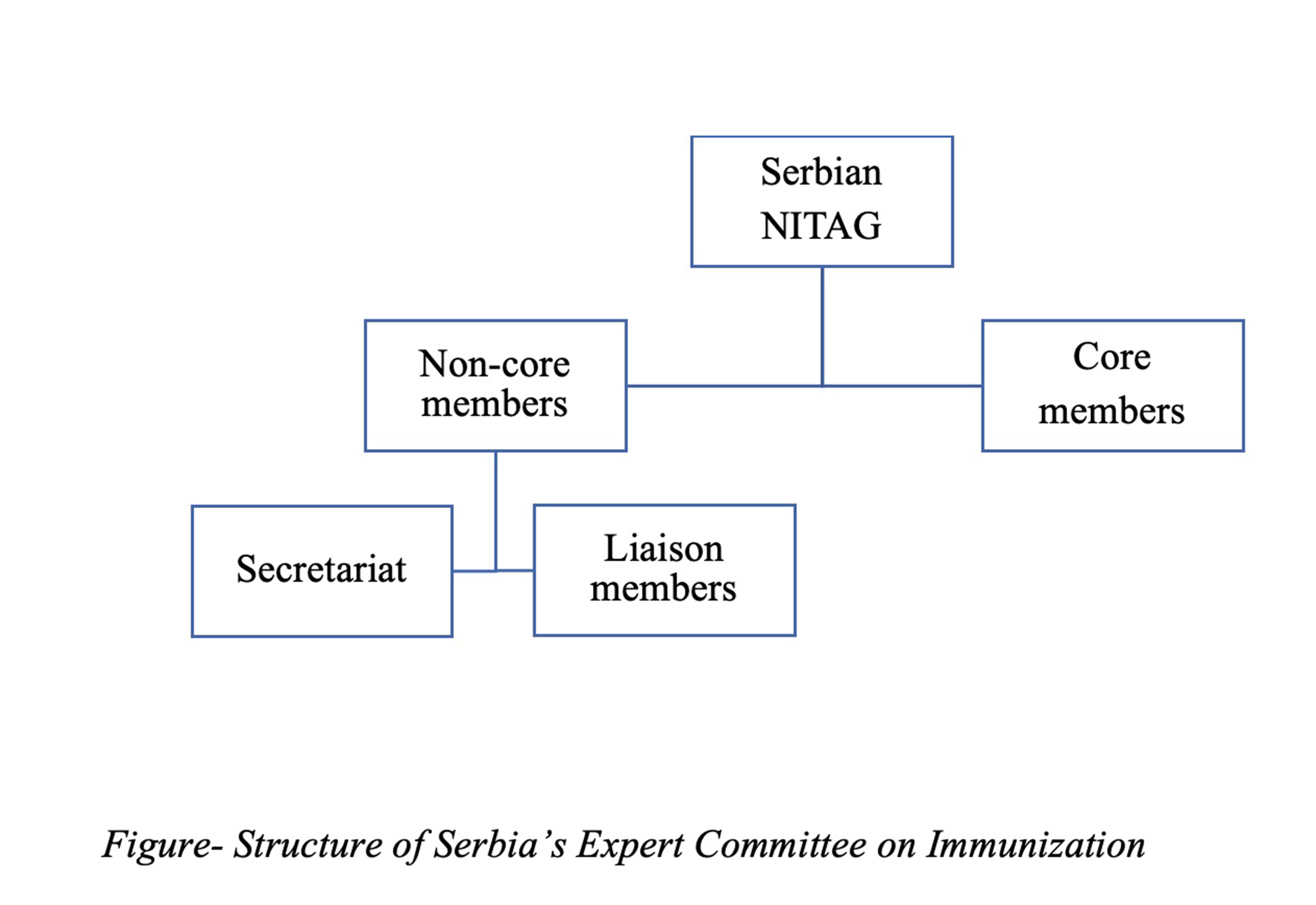

Supplement: Supplementary file 1 [file Image_1.JPEG]
